# Hydropathy

**Published:** 1844-08-10

**Authors:** 


// THE MEDICAL EXAMINER,

And Record of Medical Science.

Vol. VII.] PHILADELPHIA, SATURDAY, AUGUST 10, 1844. [No. 16.

We have received the following communication with the accompanying circular, from an esteemed correspondent. Hydropathy is the *present* reigning humbug in Europe, in the presence of which all the St. John Longs, Mesmerisers, Homœopathists, &c. are quite annihilated. Present and past experience leads us to believe that we shall soon have the epidemic here also. Many, we predict, who *now* think it most outrageous folly, or madness, before one year rolls around will lay themselves down with the utmost composure in wet sheets and bandages, quaff the cold water, and munch their stale rye bread with the greatest gusto. To the profession, however, it is of little consequence. Ignorance and credulity will ever exist, and while that is the case there will be no lack of knaves to part fools and their money.—*Ed.*

### HYDROPATHY.

To the Editor of the Medical Examiner.

This latest popular system, which seems destined to outvie homœopathy, and to flourish in its turn, until some other novelty takes its place, is now in the full tide of successful experiment in Great Britain and Europe, so far at least as money-making is concerned. The nobility and gentry are, as usual, amongst the warmest patrons of the system—and its practitioners have sagacity enough to adapt their plans to suit the purses of the wealthy. Indeed it seems to be one of the main features of all systems of quackery, to demand *high fees* for services rendered. There is no chance of success without it. It is all the fashion.

Who would go to a quack, if he asked no more for his medicine or advice than a regular physician? And the higher the demand, generally speaking, the greater the merit of the pretender in the eyes of the credulous.

The hydropathists have a fine chance of making "fat fees." The various perquisites and "et ceteras" about an establishment, will soon mount up to a good round sum. We give below a circular, handed us by a friend just returned from England, which will give some idea of the method of conducting the "water cure," in the establishment of Dr. Johnson in Hertfordshire.

*Hydropathic Establishment, Stanstead Bury House, Hertfordshire, by Dr. Johnson, author of "Life, Health, and Disease," "Theory and Principles of Hydropathy," &c., &c.*

Terms.—The terms of this Establishment vary from two guineas and a half a week upward, according to the amount of accommodation required, in apartments, &c.*

In addition to this, four shillings a week are charged for bath servants.†

Should blankets, bandages, or sheets be required for the *Treatment*, they are provided by the patients, and suitable ones may be had at the Establishment.

Those patients who have servants will be charged thirty shillings a week for the board and lodging of each—but servants can rarely be lodged *in* the Establishment, except ladies' maids.

Those who keep horses will be charged the usual terms at livery—one guinea a week for each horse.

Fires in bed rooms are charged three shillings and sixpence a week extra.

A carriage is kept for the purpose of conveying patients *to and from the railway station, on their arrival and departure.*

There being no tavern in the neighbourhood, visitors will be charged one shilling for breakfast, two shillings and sixpence for dinner, one shilling for supper, and two shillings and sixpence for a bed, (if there be a bed vacant) which monies will be received by the clerk or housekeeper, or charged to the patients visited.

Patients treated at a distance, by correspondence, are required to send a detail of symptoms every fortnight—fee one guinea each letter.

*A guinea is $5.

†An English shilling is 22 cents.

